# Supplementary material for: Knowledge, attitude, and practice of One Health and zoonotic diseases among multisectoral collaborators in Bhutan: Results from a nationwide survey
Source: PLOS Glob Public Health. 2025 Jan 9;5(1):e0004142. doi: 10.1371/journal.pgph.0004142 (PMC11717284; doi:10.1371/journal.pgph.0004142)
Supplement: S2 Table — (DOCX) [file pgph.0004142.s003.docx]

**S2 Table. Sensitivity test values**

One Health knowledge

| Variables excluded | Coefficient | Std. Error | Deviance | AIC |
| --- | --- | --- | --- | --- |
| Original model | 0.346 | 0.340 | 1233.2 | 1277.2 |
| Gender | 0.598 | 0.295 | 1244.3 | 1280.3 |
| Qualification | 0.406 | 0.308 | 1239.5 | 1277.5 |
| Professional Category | 0.498 | 0.291 | 1241.2 | 1267.2 |
| Office Level | 0.150 | 0.283 | 1235.5 | 1273.5 |
| Job Experience | 0.395 | 0.308 | 1236.9 | 1270.9 |

One Health Attitude

| Variables excluded | Coefficient | Std. Error | Deviance | AIC |
| --- | --- | --- | --- | --- |
| Original model | -0.099 | 0.333 | 1275.2 | 1319.2 |
| Gender | -0.330 | 0.322 | 1283.0 | 1325 |
| Qualification | 0.242 | 0.301 | 1281.1 | 1319.1 |
| Professional Category | 0.280 | 0.280 | 1298.9 | 1324.9 |
| Office Level | -0.078 | 0.280 | 1275.7 | 1313.7 |
| Job Experience | -0.199 | 0.301 | 1295.5 | 1329.5 |

One Health practice

| Variables excluded | Coefficient | Std. Error | Deviance | AIC |
| --- | --- | --- | --- | --- |
| Original model | 0.639 | 0.353 | 1155.4 | 1199.4 |
| Gender | 0.835 | 0.343 | 1161.5 | 1203.5 |
| Qualification | 0.512 | 0.318 | 1158.2 | 1196.2 |
| Professional Category | 0.640 | 0.294 | 1206.1 | 1232.1 |
| Office Level | 0.858 | 0.302 | 1159.8 | 1197.8 |
| Job Experience | 0.734 | 0.322 | 1160.9 | 1194.9 |

Zoonoses knowledge

| Variables excluded | Coefficient | Std. Error | Deviance | AIC |
| --- | --- | --- | --- | --- |
| Original model | 0.63877 | 0.35335 | 1155.4 | 1199.4 |
| Gender | 0.83488 | 0.34326 | 1161.5 | 1203.5 |
| Qualification | 0.51237 | 0.31811 | 1158.2 | 1196.2 |
| Professional Category | -0.51209 | 0.28160 | 1290.3 | 1316.3 |
| Office Level | -0.088779 | 0.282083 | 1255.3 | 1293.3 |
| Job Experience | 0.15851 | 0.30608 | 1269.2 | 1303.2 |

Zoonoses attitude

| Variables excluded | Coefficient | Std. Error | Deviance | AIC |
| --- | --- | --- | --- | --- |
| Original model | -0.62690 | 0.35325 | 1165.5 | 1209.5 |
| Gender | -0.58125 | 0.34164 | 1165.8 | 1207.8 |
| Qualification | -0.78029 | 0.32227 | 1167.9 | 1205.9 |
| Professional Category | -0.48001 | 0.29328 | 1188.0 | 1214.0 |
| Office Level | -0.42188 | 0.28958 | 1169.5 | 1207.5 |
| Job Experience | -0.78005 | 0.32461 | 1168.1 | 1202.1 |

Zoonoses practice

| Variables excluded | Coefficient | Std. Error | Deviance | AIC |
| --- | --- | --- | --- | --- |
| Original model | 1.04978 | 0.37694 | 1113.1 | 1157.1 |
| Gender | 1.31924 | 0.36704 | 1124.4 | 1166.4 |
| Qualification | 0.84568 | 0.34130 | 1115.6 | 1153.6 |
| Professional Category | 0.46318 | 0.29045 | 1213.9 | 1239.9 |
| Office Level | 1.40294 | 0.32585 | 1118.3 | 1156.3 |
| Job Experience | 1.11346 | 0.34594 | 1117.3 | 1151.3 |
