# Supplementary figures and images for: Knowledge, attitude, and practice of One Health and zoonotic diseases among multisectoral collaborators in Bhutan: Results from a nationwide survey
Source: PLOS Glob Public Health. 2025 Jan 9;5(1):e0004142. doi: 10.1371/journal.pgph.0004142 (PMC11717284; doi:10.1371/journal.pgph.0004142)

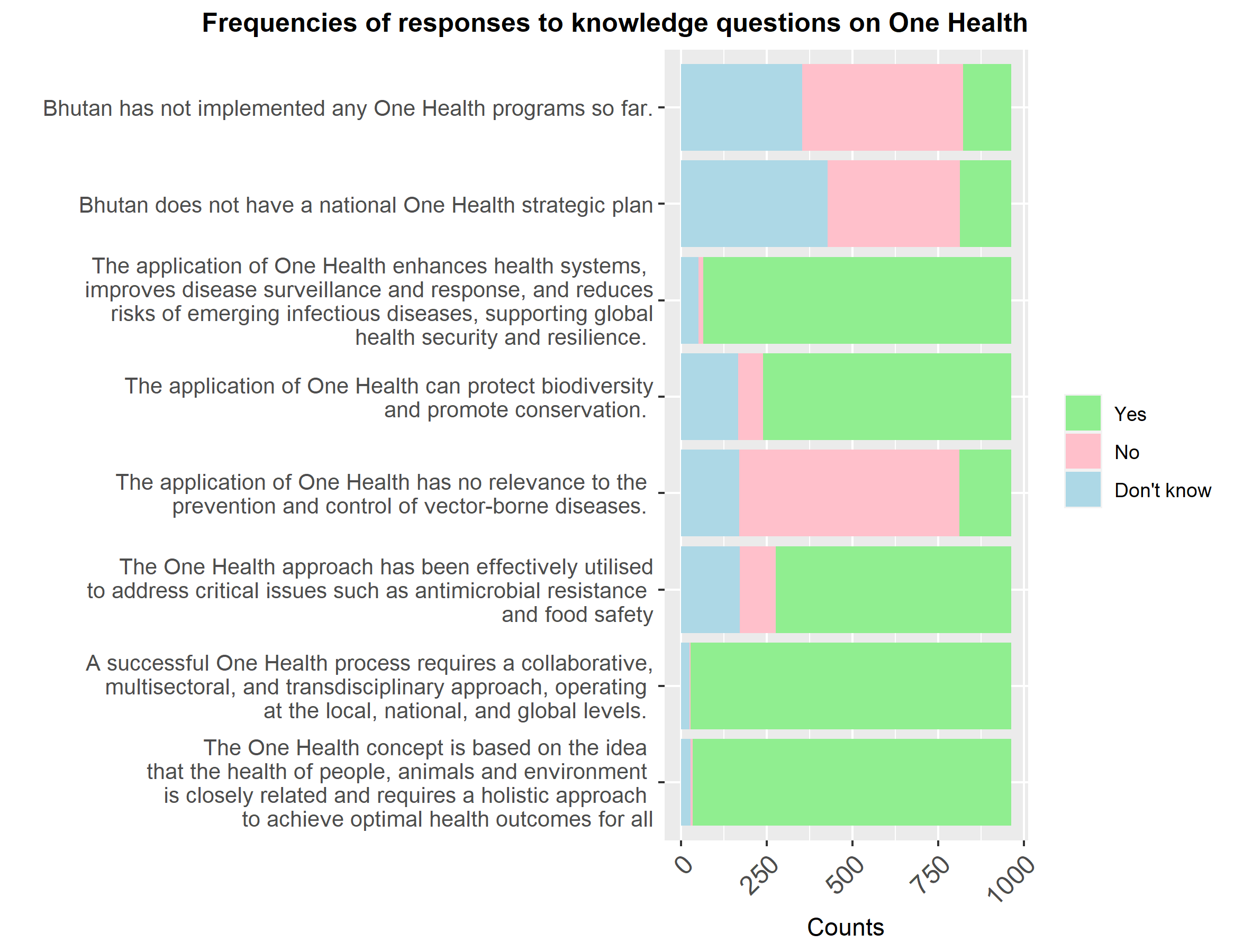

Supplement: S1 Fig — (TIF) [file pgph.0004142.s004.tif]

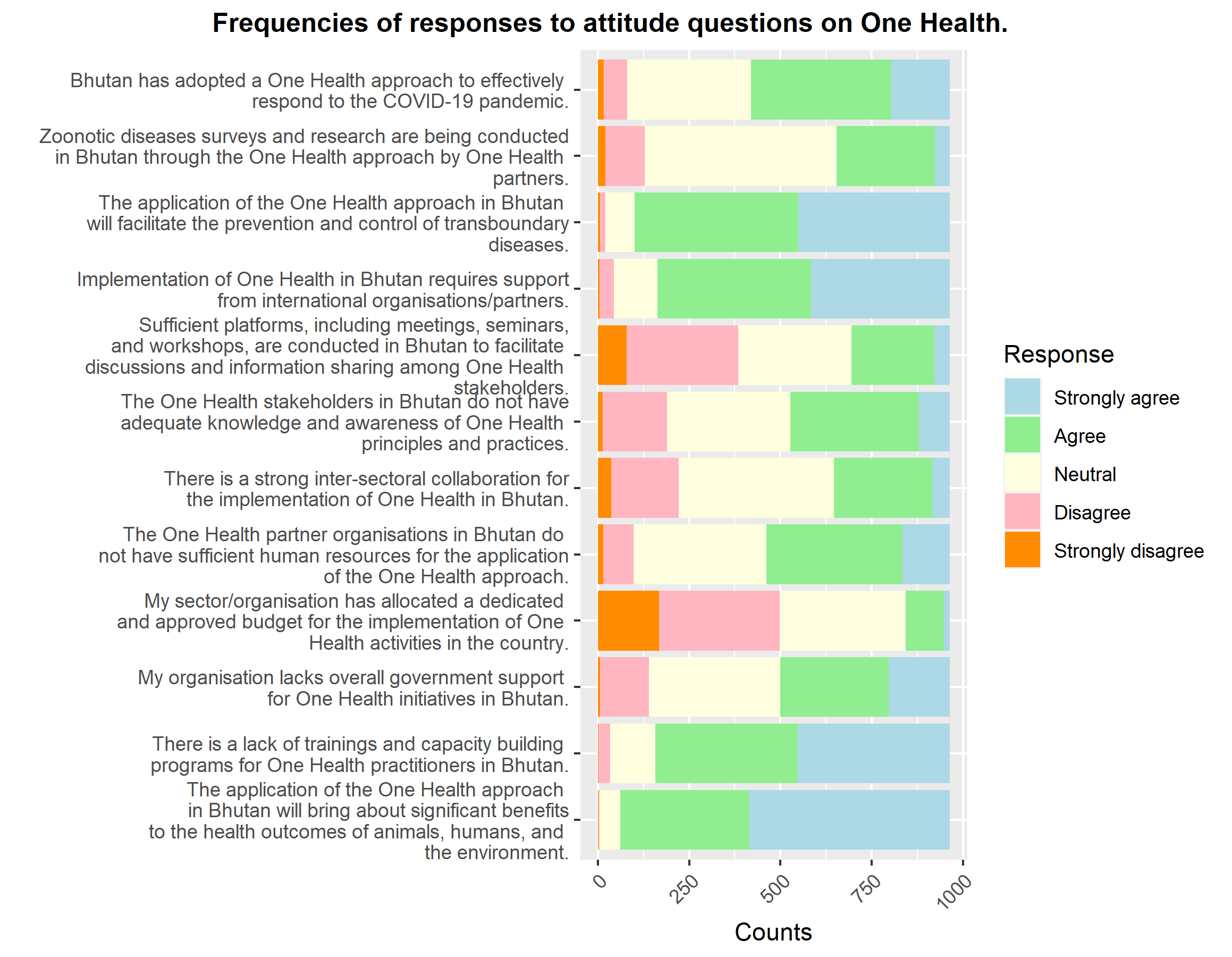

Supplement: S2 Fig — (TIF) [file pgph.0004142.s005.tif]

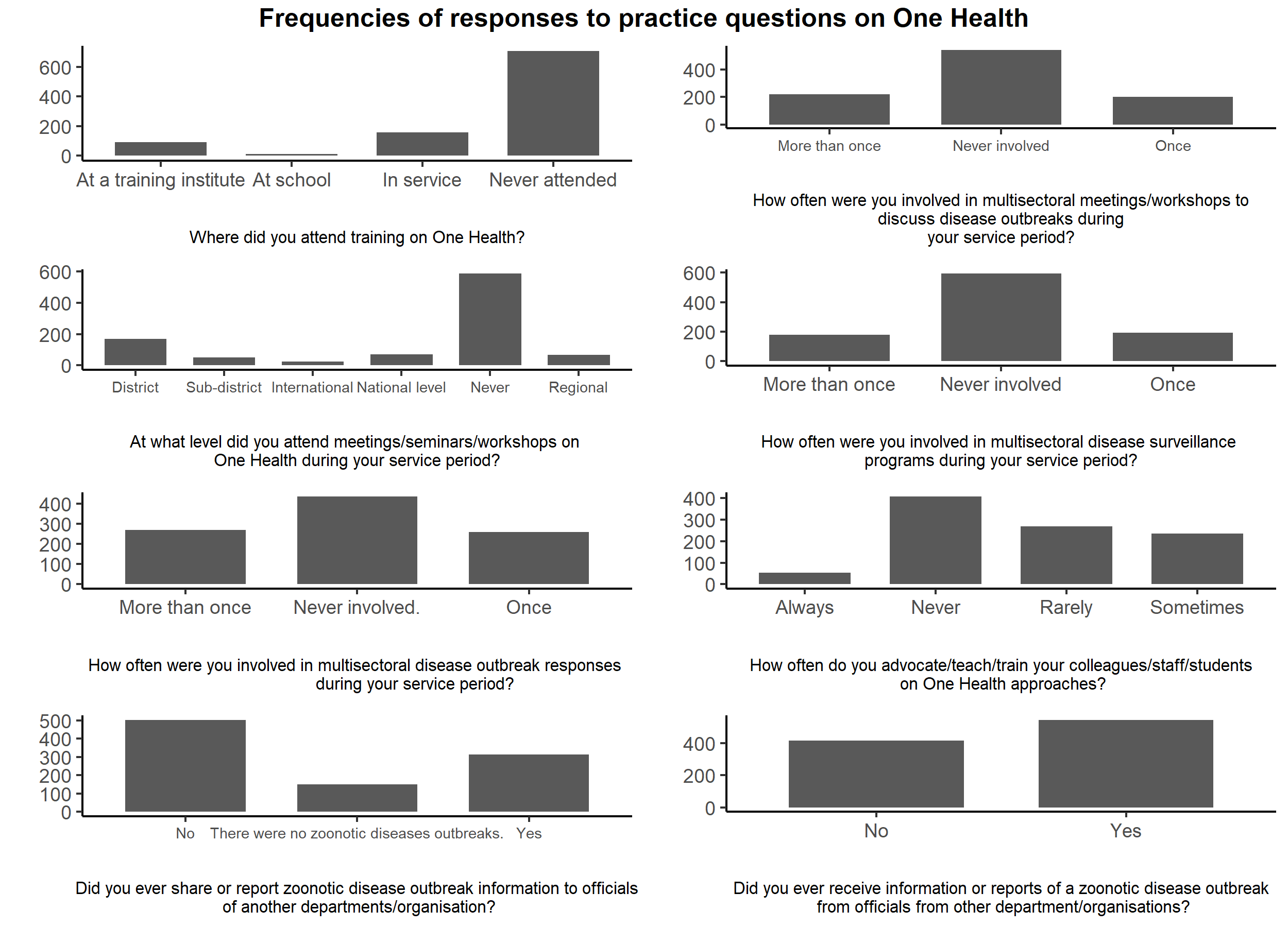

Supplement: S3 Fig — (TIF) [file pgph.0004142.s006.tif]

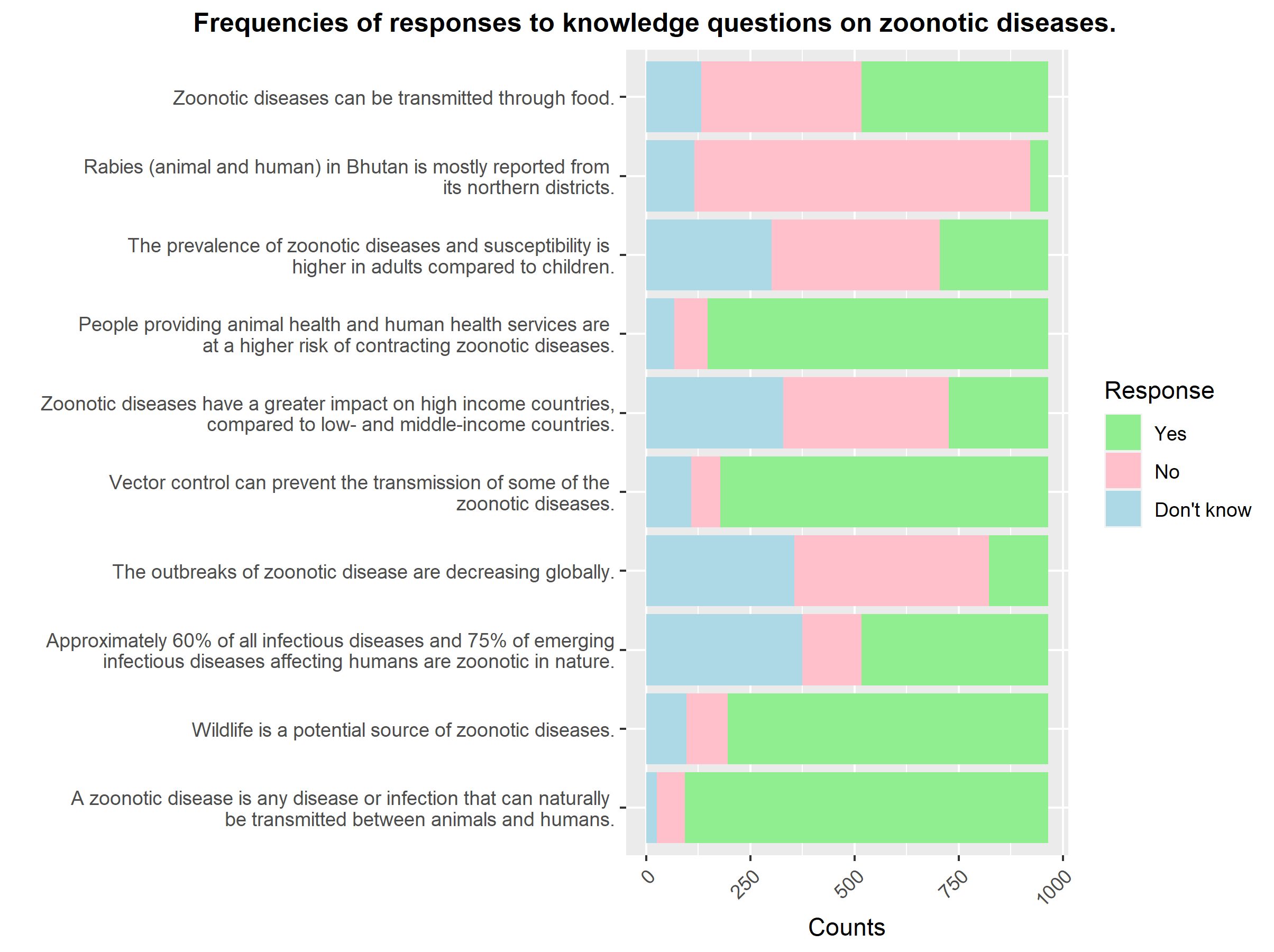

Supplement: S4 Fig — (TIF) [file pgph.0004142.s007.tif]

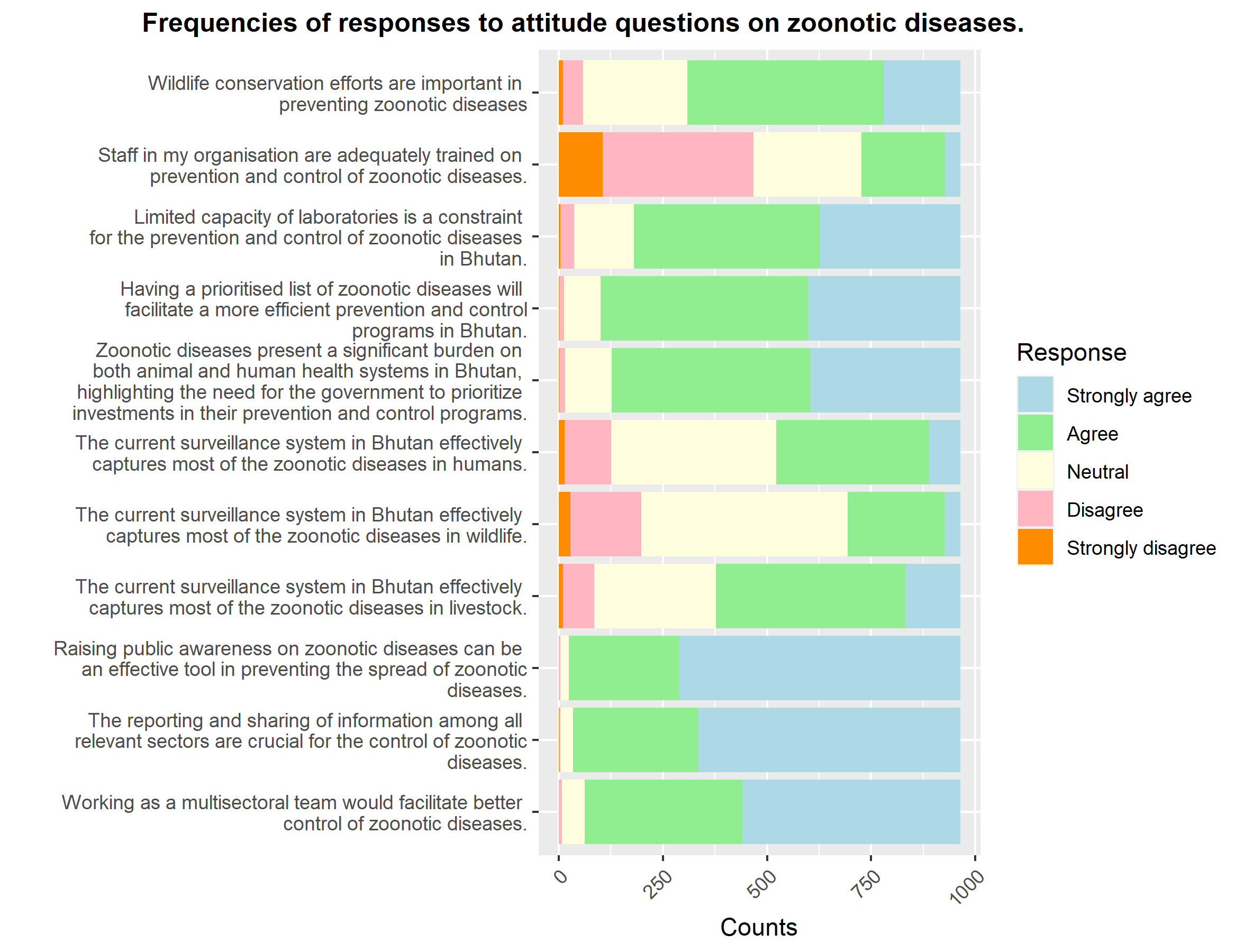

Supplement: S5 Fig — (TIF) [file pgph.0004142.s008.tif]

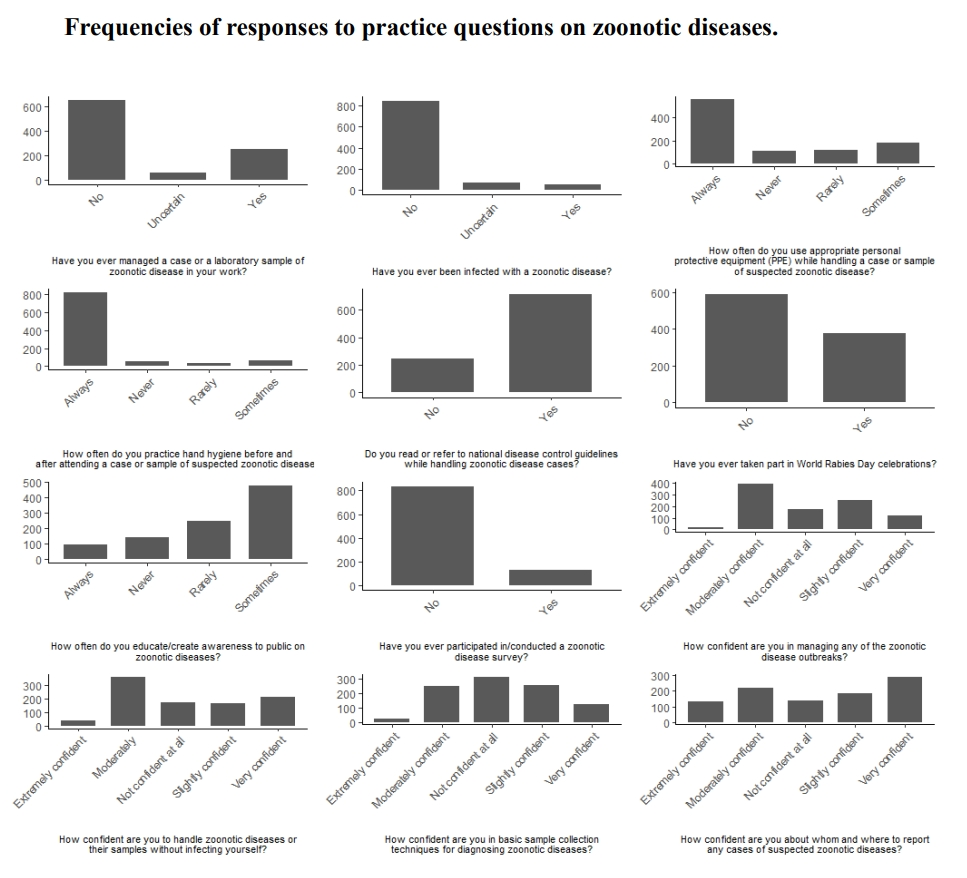

Supplement: S6 Fig — (TIF) [file pgph.0004142.s009.tif]
