# Supplementary material for: Knowledge, attitude, and practice of One Health and zoonotic diseases among multisectoral collaborators in Bhutan: Results from a nationwide survey
Source: PLOS Glob Public Health. 2025 Jan 9;5(1):e0004142. doi: 10.1371/journal.pgph.0004142 (PMC11717284; doi:10.1371/journal.pgph.0004142)
Supplement: S1 File — (DOCX) [file pgph.0004142.s010.docx]

**S1 File. Survey questionnaire**

**INFORMATION**

Dear respondent,

I am Bir Doj Rai and previously held a position in the Department of Livestock, MoAL, Bhutan. I am presently a Ph.D. student at Curtin University in Western Australia. In collaboration with Dr. Dorji Tshering, CMO, of Phuentsholing Hospital, we are conducting a survey among animal and human health workers in Bhutan.

The purpose of the survey is to assess knowledge, attitude, and practice regarding One-Health and Zoonotic diseases (diseases that can be transmitted between animals and humans) among professionals in the animal and human health sectors and professionals from other One Health sectors in Bhutan.

Considering the relevance of your position and work experience, you are invited to take part in this survey.

Participation in this research is entirely voluntary. Your decision not to participate will have no bearing on your job or any work-related evaluations or reports. You will be taken to the questionnaire section only if you consent to take part in it.

There is no immediate or direct benefit to you by participating in this research. However, the opinions you provide will help us in enhancing the application of One Health to zoonotic diseases in Bhutan, for the benefit of humans, animals, and the environment. Your opinions will be kept confidential. No personal identifiers will be attached to them. Participants will be identified by numbers for analysis. The outcome of the survey will be kept securely in soft copies with access to only the research team members.

This survey is approved by the Government of Bhutan and Curtin University through approval numbers REBH/PO /2023/007 and HRE2023-0238 respectively.

There are 69 questions in this survey. The approximate completion time required to complete the survey is 14 minutes.

**CONTACTS**

You can contact the principal researcher as below.

Bir Doj Rai, Phone No: 61 483837726.

Email: birdojrai@postgrad.curtin.edu.au

If you wish to contact co-researchers in Bhutan, please use the following details.

Dr. Dorji Tshering, CMO, Phuentsholing Hospital. Phone:+975 17517821. Email: dorjitshering@health.gov.bt

Dr.N.Dahal, Department of Livestock, MoAL. Phone: +975 2 335401 / +975 17738158. Email: ndahal@moal.gov.bt

If you wish to get more information on the research from Curtin University, please contact Professor Gavin Pereira, Curtin School of Population Health, Faculty of Health Sciences, Bentley, Perth. Phone: +61 892663940. Email: gavin.f.pereira@curtin.edu.au

**CONSENT**

Do you want to participate in this survey?

o YES

o NO

ii

**DIRECTIONS**

Please follow through the 69 survey questions and check the appropriate option that best expresses your opinion/experience. Enter text wherever asked for.

1

**Demography**

1.1 Please enter your age below (in completed years)

1.2 Whole years completed. __________________________________________________

1.2 What is your gender?

o Male

o Female

o Others

o Prefer not to say .

1.3 What is your designation? (Please enter below)

________________________________________________________________

1.4 What is the name of your office/institute? Please enter below.

________________________________________________________________

1.5 What is the level of your office / institution in terms of its service coverage?

o Gewog

o Dzongkhag

o Regional

o National

1.6 In which Dzongkhag is your office/institute located?

▼ Bumthang (1) ... Zhemgang

1.7 What is the highest level of education you have attained?

o Certificate

o Diploma

o Bachelor’s degree

o Master’s degree or above

1.8 What is the total number of years completed in your present job?

o Less than 1 year

o More than or equal to 1 year (Please enter whole completed years below)

o __________________________________________________

**2 One Health-Knowledge**

Please answer whether you agree or disagree with the following statements by selecting the appropriate options.

2.1 The One Health concept is based on the idea that the health of people, animals and environment is closely related and requires a holistic approach to achieve optimal health outcomes for all.

o Yes

o No

o Don't know.

2.2 A successful One Health process requires a collaborative, multisectoral, and transdisciplinary approach, operating at the local, national, and global levels.

o Yes

o No

o Don't know.

2.3 The One Health approach has been effectively utilised to address critical issues such as antimicrobial resistance and food safety.

o Yes

o No

o Don't know.

2.4 The application of One Health has no relevance to the prevention and control of vector-borne diseases.

o Yes

o No

o Don't know.

2.5 The application of One Health can protect biodiversity and promote conservation.

o Yes

o No

o Don't know.

2.6 The application of One Health enhances health systems, improves disease surveillance and response, and reduces risks of emerging infectious diseases, supporting global health security and resilience.

o Yes

o No

o Don't know.

2.7 Bhutan does not have a national One Health strategic plan.

o Yes

o No

o Don't know.

2.8 Bhutan has not implemented any One Health programs so far.

o Yes

o No

o Don't Know

**3 One Health- Attitude**

Please select the option that best represents your opinion for each statement below.

3.1 The application of the One Health approach in Bhutan will bring about significant benefits to the health outcomes of animals, humans, and the environment.

o Strongly agree

o Agree

o Neutral

o Disagree

o Strongly disagree.

3.2 There is a lack of training and capacity building programs for One Health practitioners in Bhutan.

o Strongly agree

o Agree

o Neutral

o Disagree

o Strongly disagree.

3.3 My organisation lacks overall government support for One Health initiatives in Bhutan.

o Strongly agree

o Agree

o Neutral

o Disagree

o Strongly disagree.

3.4 My sector/organisation has allocated a dedicated and approved budget for the implementation of One Health activities in the country.

o Strongly agree

o Agree

o Neutral

o Disagree

o Strongly disagree.

3.5 The One Health partner organisations in Bhutan do not have sufficient human resources for the application of the One Health approach.

o Strongly agree

o Agree

o Neutral

o Disagree

o Strongly disagree.

3.6 There is a strong inter-sectoral collaboration for the implementation of One Health in Bhutan.

o Strongly agree

o Agree

o Neutral

o Disagree

o Strongly disagree.

3.7 The One Health stakeholders in Bhutan do not have adequate knowledge and awareness of One Health principles and practices.

o Strongly agree

o Agree

o Neutral

o Disagree

o Strongly disagree.

3.8 Sufficient platforms, including meetings, seminars, and workshops, are conducted in Bhutan to facilitate discussions and information sharing among One Health stakeholders. -

o Strongly agree

o Agree

o Neutral

o Disagree

o Strongly disagree.

3.9 Implementation of One Health in Bhutan requires support from international organisations/partners.

o Strongly agree

o Agree

o Neutral

o Disagree

o Strongly disagree.

3.10 The application of the One Health approach in Bhutan will facilitate the prevention and control of transboundary diseases.

o Strongly agree

o Agree

o Neutral

o Disagree

o Strongly disagree.

3.11 Zoonotic diseases surveys and research are being conducted in Bhutan through the One Health approach by One Health partners.

o Strongly agree

o Agree

o Neutral

o Disagree

o Strongly disagree.

3.12 Bhutan has adopted a One Health approach to effectively respond to the COVID-19 pandemic.

o Strongly agree

o Agree

o Neutral

o Disagree

o Strongly disagree.

**4 One Health- Practice**

Please select the option that best represents your answer to the following questions.

4.1 Where did you attend training on One Health?

o At school

o At a training institute

o In service

o Never attended.

4.2 How often were you involved in a multisectoral meetings/workshops to discuss about diseases outbreaks during your service period?

o More than once

o Once

o Never involved

4.3 At what level did you attend meetings/seminars/workshops on One Health during your service period? (Mention the highest level if you have attended multiple meetings)

o International level

o National level

o Regional level

o District level.

o Gewog level

o Never attended.

4.4 How often were you involved in multisectoral disease surveillance programs during your service period?

o More than once

o Once

o Never involved.

4.5 How often were you involved in multisectoral disease outbreak responses during your service period?

o More than once

o Once

o Never involved.

4.6 How often do you advocate/teach/ train your colleagues/staff/students on One Health approaches

o Always

o Sometimes

o Rarely

o Never.

4.7 Did you ever share or report zoonotic disease outbreak information to officials of another departments/organisation?

o Yes

o No

o There were no zoonotic diseases outbreaks.

4.8 Did you ever receive information or reports of a zoonotic disease outbreak from officials from other department/organisations?

o Yes

o No

**5 Zoonotic diseases- Knowledge.**

Please answer whether you agree or disagree with the following statements by selecting the appropriate options.

5.1 A zoonotic disease is any disease or infection that can naturally be transmitted between animals and humans.

o Yes

o No

o Don't know.

5.2 Wildlife is a potential source of zoonotic diseases.

o Yes

o No

o Don't know .

5.3 Approximately 60% of all infectious diseases and 75% of emerging infectious diseases affecting humans are zoonotic in nature.

o Yes

o No

o Don't know.

5.4 The outbreaks of zoonotic disease are decreasing globally.

o Yes

o No

o Don't know.

5.5 Vector control can prevent the transmission of some of the zoonotic diseases.

o Yes

o No

o Don't know.

5.6 Zoonotic diseases have a greater impact on high income countries, compared to low- and middle-income countries.

o Yes

o No

o Don't know.

5.7 People providing animal health and human health services are at a higher risk of contracting zoonotic diseases.

o Yes

o No

o Don't know.

5.8 The prevalence of zoonotic diseases and susceptibility is higher in adults compared to children.

o Yes

o No

o Don't know.

5.9 Rabies (animal and human) in Bhutan is mostly reported from its northern districts.

o Yes

o No

o Don't know.

5.10 Zoonotic diseases can be transmitted through food.

o Yes

o No

o Don't know.

**6 Zoonotic diseases- Attitude**

Please select the option that best represents your opinion for each statement below.

6.1 Working as a multisectoral team would facilitate better control of zoonotic diseases.

o Strongly agree

o Agree

o Neutral

o Disagree

o Strongly disagree.

6.2 The reporting and sharing of information among all relevant sectors are crucial for the control of zoonotic diseases.

o Strongly agree

o Agree

o Neutral

o Disagree

o Strongly disagree.

6.3 Raising public awareness on zoonotic diseases can be an effective tool in preventing the spread of zoonotic diseases.

o Strongly agree

o Agree

o Neutral

o Disagree

o Strongly disagree.

6.4 The current surveillance system in Bhutan effectively captures most of the zoonotic diseases in livestock.

o Strongly agree

o Agree

o Neutral

o Disagree

o Strongly disagree.

6.5 The current surveillance system in Bhutan effectively captures most of the zoonotic diseases in wildlife.

o Strongly agree

o Agree

o Neutral

o Disagree

o Strongly disagree.

6.6 The current surveillance system in Bhutan effectively captures most of the zoonotic diseases in humans.

o Strongly agree

o Agree

o Neutral

o Disagree

o Strongly disagree.

6.7 Zoonotic diseases present a significant burden on both animal and human health systems in Bhutan, highlighting the need for the government to prioritise investments in their prevention and control programs.

o Strongly agree

o Agree

o Neutral

o Disagree

o Strongly disagree.

6.8 Having a prioritised list of zoonotic diseases will facilitate a more efficient prevention and control programs in Bhutan.

o Strongly agree

o Agree

o Neutral

o Disagree

o Strongly disagree.

6.9 Limited capacity of laboratories is a constraint for the prevention and control of zoonotic diseases in Bhutan.

o Strongly agree

o Agree

o Neutral

o Disagree

o Strongly disagree.

6.10 Staff in my organisation are adequately trained on prevention and control of zoonotic diseases.

o Strongly agree

o Agree

o Neutral

o Disagree

o Strongly disagree.

6.11 Wildlife conservation efforts are important in preventing zoonotic diseases

o Strongly agree

o Agree

o Neutral

o Disagree

o Strongly disagree.

**7 Zoonotic diseases- Practice**

Please select the option that best represents your answer to the following questions.

7.1 Have you ever managed a case or a laboratory sample of zoonotic disease in your work?

o Yes

o No

o Uncertain.

7.2 Have you ever been infected with a zoonotic disease?

o Yes

o No

o Uncertain.

7.3 How often do you use appropriate personal protective equipment (PPE) while handling a case or sample of suspected zoonotic disease

o Always

o Sometimes

o Rarely

o Never.

7.4 How often do you practice hand hygiene before and after attending a case or sample of suspected zoonotic disease?

o Always

o Sometimes

o Rarely

o Never .

7.5 Do you read or refer to national disease control guidelines while handling zoonotic disease cases?

o Yes

o No .

7.6 Have you ever taken part in World Rabies Day celebrations?

o Yes

o No

7.7 How often do you educate / create awareness to public on zoonotic diseases?

o Always

o Sometimes

o Rarely

o Never.

7.8 Have you ever participated in a / conducted a zoonotic disease survey?

o Yes

o No.

7.9 How confident are you in managing any of the zoonotic disease outbreaks?

o Extremely confident

o Very confident

o Moderately confident.

o Slightly confident

o Not confident at all.

7.10 How confident are you to handle zoonotic diseases or their samples without infecting yourself?

o Extremely confident

o Very confident

o Moderately

o Slightly confident

o Not confident at all.

7.11 How confident are you in basic sample collection techniques for diagnosing zoonotic diseases?

o Extremely confident

o Very confident

o Moderately confident

o Slightly confident

o Not confident at all.

7.12 How confident are you about whom and where to report any cases of suspected zoonotic diseases?

o Extremely confident

o Very confident

o Moderately confident

o Slightly confident

o Not confident at all.
