# Supplementary material for: Knowledge, attitude, and practice of One Health and zoonotic diseases among multisectoral collaborators in Bhutan: Results from a nationwide survey
Source: PLOS Glob Public Health. 2025 Jan 9;5(1):e0004142. doi: 10.1371/journal.pgph.0004142 (PMC11717284; doi:10.1371/journal.pgph.0004142)
Supplement: Attachment — Submitted filename: Reviewers comments.docx [file pgph.0004142.s011.docx]

**Reviewer’s Comments**

The manuscript provides a comprehensive and well-organized overview of survey results concerning knowledge, attitudes, and practices (KAP) related to One Health (OH) among multisectoral respondents in Bhutan. It effectively highlights the significance of enhancing OH strategies in the country. With minor revisions to improve clarity and depth, the paper holds potential as a robust framework for advocating necessary changes in veterinary public health policy.

Outlined below are the strengths of the paper, followed by recommendations for improvement of manuscript:

**Strengths of the Paper:**

**Clarity of Purpose**: The study's objective is clearly articulated, aiming to assess the knowledge, attitudes, and practices regarding OH and zoonotic diseases among professionals in Bhutan.

**Identification of Gaps**: The paper concisely identifies key gaps, including insufficient training, limited funding, lack of research, low awareness of OH programs, and inadequate cross-sectoral collaboration. This is crucial for understanding the current OH scenario in Bhutan.

**Statistics and Data**: The inclusion of detailed statistics effectively illustrates the level of OH knowledge among respondents, highlighting distinctions by gender and educational qualification.

**Actionable Recommendations**: The paper provides constructive and practical recommendations, particularly the need for targeted educational interventions and improved communication strategies.

**Broader Implications**: The study’s findings are framed within a global context, offering insights relevant to other low- and middle-income countries, which adds significant value to the paper.

**Recommendations/Suggestions to improve the manuscript**

**Sentence Structure**: Some of the complex sentences in the paper could be simplified to improve readability. Breaking down these sentences into shorter, more digestible parts would enhance clarity for the readers.

**Acronym Definition**: A brief explanation of "One Health" should be provided for readers unfamiliar with the term. Additionally, terms like "AOR" (Adjusted Odds Ratio) and "CI" (Confidence Interval) require brief definitions to ensure clarity for those not well-versed in statistical terminology.

**Clarification of Data**: The term "above-average knowledge" should be defined more explicitly to provide greater meaning and context at the beginning of the discussion.

**Discussion of Implications**: Consider including a discussion on the potential impact of the findings on OH policy or public health practice. This would underscore the practical significance of the study.

**Methodology Details**: Providing a brief explanation of the sample size calculation would strengthen the study's credibility. Additionally, the rationale for conducting this KAP survey should be elaborated, emphasizing its importance.

**Introduction Section**: The introduction could benefit from a more detailed and scientifically rigorous description of the gaps and challenges identified in the study.

**Flow and Transition:** The transition between the identification of gaps and the subsequent recommendations could be smoother. A clearer connection between the identified gaps and the proposed interventions would enhance the paper’s coherence.

**Examples of Implementation**: Including examples of successful OH strategies from other regions could provide stronger support for the recommendations made in the study.

**Citations and References**: The reference to different figures should be accompanied by brief descriptions to provide context. This would help readers understand the relevance of these figures within the broader study.

**Connection Between Findings**: Strengthening the link between attitudes and the need for training or support is recommended. For example, discussing how attitudes may influence the demand for OH training could add depth to the findings.

By addressing these suggestions, the paper could significantly improve its overall impact and contribution to the field of One Health.
