# Supplementary material for: Knowledge, attitude, and practice of One Health and zoonotic diseases among multisectoral collaborators in Bhutan: Results from a nationwide survey
Source: PLOS Glob Public Health. 2025 Jan 9;5(1):e0004142. doi: 10.1371/journal.pgph.0004142 (PMC11717284; doi:10.1371/journal.pgph.0004142)
Supplement: Attachment — Submitted filename: Review_Plos Global Health_Oct 23, 2024.docx [file pgph.0004142.s012.docx]

**Review (Oct 23, 2024)**

Manuscript #: PGPH-D-24-01911

**Title:** Knowledge, attitude, and practice of One Health and zoonotic disease among multi-sectorial collaborators in Bhutan: Results from a nationwide survey

**Authors:** Bir Doj Rai; Tenzin Tenzin; Dorji Tshering; Narapati Dahal; Gizachew A Tessema; Lin Fritschi; Sylvester Nyandu Dodzi; Gavin Pereira

**Article type:** Research Article

**Review results:**

Thank you so much for sharing this cross-sectional, facility-based study that was carried out nationwide through an online questionnaire survey in Bhutan. The topic is very interesting and useful for other countries and regions related to One Health (OH). There are some points that I would like to suggest to improve this manuscript's quality.

**Overall suggestions:**

Please check for grammatical errors, including tenses, citation formats, and spaces between texts throughout the manuscript. Instead of simply referencing previous studies, study methodologies should be more detailed.

**Title:**

Should we change to "zoonotic diseases" instead of "zoonotic disease"?

**Abstract:**

- This study assesses the knowledge, attitudes, and practices regarding One Health and zoonotic diseases among key sector professionals to identify gaps and opportunities for enhancing One Health strategies in Bhutan. Please change to "assessed".
- …..and two-thirds (66%) had above-average 32 practices. Professionals with mid-level job experience ( 6-10 years; AOR = 2.13; 95% CI = 33 1.37-3.30 and 11 to 15 years; AOR = 2.11; 95% CI = 1.31-3.40). Please change to (6-10 years; …)

**Introduction:**

- Page 3, Line 55: Please revise to "OH concept" has proven …..
- Page 3, Line 57: Please provide more information about the drivers and impacts of zoonotic diseases in Bhutan that could be reasons for forming OH, aside from having a list of zoonotic diseases (Page 4, Line 60 – 62). Kindly provide information, especially national health statistics on these zoonotic diseases.
- Page 4, Line 70: Please provide more information about having anthrax, rabies, avian influenza, antimicrobial resistance, and leptospirosis[26], as priority diseases.
- Page 4, Line 73: please provide more information about sources of external funding.
- Page 4, Line 73 – 75: please elaborate on the challenges.
- Page 4, Line 75 – 76: please elaborate on "requires behavioural, attitudinal and institutional change".
- Page 4, Line 76 – 78: Please elaborate on these sentences "A Knowledge, Attitude and Practice (KAP) study among key stakeholders can identify opportunities and challenges, providing context-specific information essential to inform and evaluate OH strategies for Bhutan and globally.[27]. Moreover, delete . after globally.
- Page 4 - 5, Line 81 – 85: Are these the current results? If not, please provide citations. If yes, please move to the result section.
- Page 5, Line 86: Please change to "This research results are expected to contribute ….."

**Methodology:**

- Page 5, Line 90 – 99: Please check the Plos format to see whether the "Ethics statement" should be placed here. Please also provide information on how you reached the target respondents for this online survey; for example, how you got their email addresses or shared the online questionnaire.
- Page 6, Line 106 – 116: Please confirm and provide information on whether they have been involved with OH activities.
- Please revise the sequences of this section per the journal's format. I would suggest the following sequences: Study design, Study locations, Study respondent selections, data collection procedures, and data analysis.
- Please add a sub-section to briefly explain Bhutan's health (human and animal) systems and administration. Then, the readers can understand the rationale behind selecting the professions.
- Page 7, Line 122: please change "invitees" to "respondents or participants."
- Page 7, Line 122 – 126: please provide a rationale for selecting these professions for this study.
- Page 7, Line 137 – 138: Please provide information about the pre-testing among the respondents. Did the pre-test results were included in the actual analysis?
- Page 8, Line 142: how did you obtain their email addresses? Did they agree to share their email addresses with the research team?
- Page 8, Line 144: What data collection strategies were used to get a high response rate within a very short period (September 18 to October 13, 2023)? Did you share the online questionnaire with potential respondents during the nationwide meetings?
- Page 8, Line 150: Please elaborate on the scoring methods that were similar to the previous studies (ref. 31 and 32).
- Page 8, Line 157: Please elaborate on methods instead of only mentioning, “We followed methods used in other studies [36, 37]”.
- As per the abstract – please elaborate: “Responses from participants were categorized into binary outcomes based on the mean score. Descriptive and summary statistics were performed.”, in the analysis methods of the methodology section.

**Results:**

- Page 9, Line 180 – 181: Please check (Table 1) which was separated into different lines.
- Table 1: Please revise the format of the Table.
- Page 11, Line 184: Please add – Remark: BFDA, Bhutan Food and Drug Authority
- Table 2 - 3: Please revise the tables’ formats and presentation. Also, Table 2, please correct the table headings are correct, which columns are AOR, and which are 95% CI.

**Discussions:**

- Page 22, Line 257: Please change to “demonstrated”.
- Page 22, Line 257 – 259: Please paraphrase these sentences.
- Page 23, Line 285: Please provide study locations of the previous studies (ref 32 and 44) instead of only mentioning the “similar studies”.
- Page 23, Line 288 – 289: Please elaborate on “the needs” in specific activities and curricula, such as knowledge of topics that should be improved, such as clinical, epidemiology, OH, or else?
- Page 24, Line 304 – 308: Please elaborate on the results of the previous studies compared with the current findings.
- Page 24, Line 321 – 324: Please elaborate on the rationale for the limited number of studies in Bhutan that could be associated with the current results, including the OH implementation in the country.
- Any strengths of this current study?
- As mentioned, the limitation of the study was the exclusion of professionals from the environmental sector. Kindly elaborate on additional limitations of other selection biases, such as selecting only those who have email addresses or were pre-selected. As the questionnaire was quite lengthy, how could you avoid missing responses per question? Did you include all the missing items in the analysis?
- Please have a separate “recommendation section” and provide in-depth plans and strategies that policymakers should consider to improve the OH structure and implementation activities.

**Conclusions:**

- Page 25, Line 336: Please change to “highlighted” instead of “highlights”.
- Page 25, Line 337: Please revise the wording from “deficiencies” to another term.
