# Supplementary material for: Knowledge, attitude, and practice of One Health and zoonotic diseases among multisectoral collaborators in Bhutan: Results from a nationwide survey
Source: PLOS Glob Public Health. 2025 Jan 9;5(1):e0004142. doi: 10.1371/journal.pgph.0004142 (PMC11717284; doi:10.1371/journal.pgph.0004142)
Supplement: Attachment — Submitted filename: Response to Reviewers.docx [file pgph.0004142.s013.docx]

**Knowledge, attitude, and practice of One Health and zoonotic diseases among multisectoral collaborators in Bhutan: Results from a nationwide survey**

**RESPONSES TO REVIEWS**

**Reviewers**

Reviewer 1

Reviewer 2

Bir Doj Rai^1*^, Tenzin Tenzin ^2&^, Dorji Tshering^3&^, Narapati Dahal^1&^, Gizachew A Tessema,^4,#&^ Lin Fritschi,^4&^ Sylvester Nyandu Dodzi^4&^, Gavin Pereira^4,#&^

^1^Department of Livestock, Ministry of Agriculture and Livestock, Thimphu, Bhutan.

^2^ Sub-Regional Representation for Southern Africa, World Organisation for Animal Health, Gaborone, Botswana.

^3^Phuentsholing General Hospital, Ministry of Health, Chhukha, Bhutan.

^4^Curtin School of Population Health, Curtin University, Perth, Western Australia.

#enAble Institute, Curtin University, Perth, Western Australia.

* Corresponding author

E-mail: [birdoj_rai@yahoo.com](mailto:birdoj_rai@yahoo.com) (BDR)

# Reviewer 1

## Overall comments

The manuscript provides a comprehensive and well-organized overview of survey results concerning knowledge, attitudes, and practices (KAP) related to One Health (OH) among multisectoral respondents in Bhutan. It effectively highlights the significance of enhancing OH strategies in the country. With minor revisions to improve clarity and depth, the paper holds potential as a robust framework for advocating necessary changes in veterinary public health policy.

Outlined below are the strengths of the paper, followed by recommendations for improvement of manuscript:

## Strengths of the Paper

- **Clarity of Purpose:** The study's objective is clearly articulated, aiming to assess the knowledge, attitudes, and practices regarding OH and zoonotic diseases among professionals in Bhutan.
- **Identification of Gaps:** The paper concisely identifies key gaps, including insufficient training, limited funding, lack of research, low awareness of OH programs, and inadequate cross-sectoral collaboration. This is crucial for understanding the current OH scenario in Bhutan.
- **Statistics and Data:** The inclusion of detailed statistics effectively illustrates the level of OH knowledge among respondents, highlighting distinctions by gender and educational qualification.
- **Actionable Recommendations:** The paper provides constructive and practical recommendations, particularly the need for targeted educational interventions and improved communication strategies.
- **Broader Implications:** The study’s findings are framed within a global context, offering insights relevant to other low- and middle-income countries, which adds significant value to the paper.

**Response:** We sincerely appreciate your time and effort in reviewing our manuscript and acknowledging its strengths.

**Recommendations/Suggestions to improve the manuscript**

**Comment 1. Sentence Structure:** Some of the complex sentences in the paper could be simplified to improve readability. Breaking down these sentences into shorter, more digestible parts would enhance clarity for the readers.

**Response:** Thank you for your constructive feedback. We have now thoroughly reviewed the manuscript and simplified the complex statements to enhance readability and ensure clarity for all readers.

**Comment 2. Acronym Definition:** A brief explanation of "One Health" should be provided for readers unfamiliar with the term. Additionally, terms like "AOR" (Adjusted Odds Ratio) and "CI" (Confidence Interval) require brief definitions to ensure clarity for those not well-versed in statistical terminology.

**Response:** Thank you. We have now included a brief explanation on One Health in our introduction, as follows:

Page 3, Lines 42 – 45*: “One Health (OH) is an integrated approach that aims to optimise the health of people, animals, and ecosystems by recognising their interconnectedness [1]. It employs a coordinated, collaborative, multidisciplinary, and cross-sectoral strategy to manage risks originating at the animal-human-ecosystem interface [2].”*

Furthermore, we have defined the statistical terminologies, AOR (Adjusted Odds Ratio) and CI (Confidence Interval) in our Materials and methods (Data analysis) section as follows:

Page 11, Lines 217 – 221: *“An AOR (Adjusted Odds Ratio) indicates the strength of the relationship between one factor and an outcome while accounting for the effects of other factors [3]. The 95% CI (Confidence Interval) represents the range within which the true value of the AOR is expected to fall 95% of the time, providing insight into the reliability and precision of the estimate [4].”*

**Comment 3.** Clarification of Data: The term "above-average knowledge" should be defined more explicitly to provide greater meaning and context at the beginning of the discussion.

**Response:** As suggested, we have now expanded our definition for “above-average” and “below-average”.

Page 10, Lines 204 – 208: *“In this study, we defined respondents in the “above-average” category as those scoring at or above the mean on questions related to knowledge, attitudes and practices , indicating higher levels in these areas compared to the average respondent in our study population. Conversely, the “below -average” category referred to those respondents scoring below the mean, representing lower levels of knowledge, attitudes and practices.”*

**Comment 4.** Discussion of Implications: Consider including a discussion on the potential impact of the findings on OH policy or public health practice. This would underscore the practical significance of the study.

**Response:** Thank you for your insightful suggestion. We have now added a recommendation section in the manuscript. This section includes practical strategies and actionable recommendations to enhance One Health policies and practices.

The recommendations section is shown below: (Pages 26 – 28, Lines 436 – 486)

***Recommendations***

*To address the key challenges identified in our study, we propose the following recommendations to enhance OH practices in Bhutan. Although tailored to Bhutan’s context, these recommendations hold broader global relevance, as similar challenges in OH implementation are prevalent across many LMICs [5]. These strategies provide actionable insights that can be adapted to strengthen OH systems in comparable settings worldwide.*

***Strengthen communication and awareness:*** *We recommend strengthening communication and awareness on OH and zoonotic diseases among all stakeholders, by developing a comprehensive communication strategy. The strategic communication framework of the World Health Organisation [6], with its wide intended audiences in health settings and actionable guidance, serves as a valuable resource for formulating such communication strategies.*

*Additionally, regular intersectoral meetings among stakeholders will help align operational activities with strategic objectives, facilitating effective integration at all levels.*

***Develop One Health workforce:*** *We recommend conducting workforce mapping to identify human resource gaps and inform both short and long-term capacity development strategies.* *Short-term strategies should focus on joint in-service training on foundational OH concepts such as collaboration and communication, progressing to skills in zoonotic disease management, field epidemiology, laboratory technologies, and policy formulation. Long-term strategies should prioritise integrating OH education into CNR and KGUMSB curricula, ensuring competencies among future OH professionals. Curriculum development can draw on models such as those developed by the One Health Central and Eastern Africa (OHCEA) network [7] and the FAO [8]. Such efforts may be regularly evaluated using frameworks such as the OH core competencies [9] to identify the areas of improvement and enhance efficiency*

***Enhance collaboration:*** *We recommend strengthening intersectoral and international collaboration among key OH stakeholders to improve the OH system’s efficiency. Regular meetings involving all stakeholders can foster collaboration, leading to integrated initiatives such as joint trainings, surveillance systems, and action plans. The outcomes form the International Health Regulations-Performance of Veterinary Services National Bridging Workshop [10] may be utilised to prioritise key areas of collaboration for enhancement between human and animal health sectors. Furthermore, the Bhutan OH Secretariat should actively engage with international organisations to increase visibility of Bhutan’s commitment to OH, through networking, and attract further support for OH initiatives.*

***Effectively utilise disease prioritisation outcomes:*** *Bhutan has prioritised zoonotic diseases using a credible tool employed in multiple countries [11]. We recommend applying these insights to direct investment in prevention and control strategies employing OH approaches. Furthermore, risk assessments of the prioritised zoonoses will be highly beneficial for guiding in developing guidelines, protocols, and strengthening laboratory capacity to manage zoonotic risks.*

***Allocate adequate resources:*** *We recommend policy advocacy and reforms to facilitate sustainable funding and structural support for OH initiatives. Among other priorities, increased funding is recommended in areas such as human capital building, laboratory capacity development, research, surveillance systems and intersectoral coordination. Furthermore, pooling resources across sectors is recommended to prevent redundancy and maximise the impact of available resources.*

***Enhance research:*** *We recommend enhancing research in areas of zoonotic diseases and policy issues, through training, networking, and multidisciplinary collaboration to inform technical and policy decisions for OH initiatives. Furthermore, establishing a comprehensive research framework can streamline efforts, promote coordination, and ensure evidence-based approaches to addressing OH challenges.*

**Comment 5.** **Methodology Details:** Providing a brief explanation of the sample size calculation would strengthen the study's credibility. Additionally, the rationale for conducting this KAP survey should be elaborated, emphasizing its importance.

**Response:** To address the concern regarding sample size calculation, we have explained our sample size calculation in Materials and methods (Respondent selection) section as follows:

Page 9, Lines 164 – 167: *“We aimed to recruit as many eligible professionals as possible, with a minimum target sample size of 385. This target was calculated based on an assumed baseline proportion of 50% for adequate knowledge, attitudes, and practices, a 5% margin of error, and a 95% confidence level (Z=1.96).”*

We have also stated the rationale and importance of KAP (Knowledge, Attitudes, and Practices) study in our in our introduction:

Page 5, Lines 98 – 106*: “**Addressing these challenges in OH requires behavioural, attitudinal, and institutional changes [12]. This involves accepting a culture of collaboration and communication among OH stakeholders, shifting attitudes towards shared responsibility for health outcomes, and policy alignment across sectors [13].* *A Knowledge, Attitude and Practice (KAP) study among key stakeholders can identify opportunities and challenges that influence the success of OH strategies. By assessing the current level of awareness, perceptions and attitudes related to OH and zoonotic diseases among the stakeholders, such a study provides context-specific information that is essential for making targeted interventions [14].”*

**Comment 6.** **Introduction Section:** The introduction could benefit from a more detailed and scientifically rigorous description of the gaps and challenges identified in the study.

**Response**: Thank you for the suggestion. We have elaborated on the challenges and gaps as follows:

Page 5, Lines 90 – 98: *“Despite these initiatives, significant challenges hinder OH efforts to control zoonotic diseases in Bhutan, and there is limited national comprehensive scientific inquiry to identify the challenges and possible remedies. Major challenges include inadequate diagnostic capacity due to limited government funding, poorly defined roles of stakeholders, and a lack of formal collaborative mechanisms [15]. Furthermore, surveillance and response measures are weak due to insufficient collaboration among key stakeholders [15]. Limited data for evidence-based decision-making, due to a scarcity of localised and policy-relevant research, presents an additional challenge [12, 16].”*

**Comment 7. Flow and Transition:** The transition between the identification of gaps and the subsequent recommendations could be smoother. A clearer connection between the identified gaps and the proposed interventions would enhance the paper’s coherence.

**Response:** Thank you for this valuable feedback. We have carefully reviewed the sections with unclear connections and have restructured them to create smoother transitions and clearer links between the identified gaps and the proposed interventions in the manuscript.

Examples 1. Page 24, Lines 368 – 372: *“The administrators in our study, demonstrated above-average practices, likely due to their access to OH resources, decision-making authority and, exposure to OH initiatives. Their strategic roles position them as key leaders for OH programmes at the district level. Therefore, strengthening administrators’ engagement and leadership could further enhance the effectiveness of OH initiatives at the local level.”*

Example 2. Page 25, Lines 393 – 400: “*The below-average attitudes observed among half of the respondents coupled with the identified need for prioritising investment suggest a need to improve resource allocation. To address this need, the government may benefit from considering the outcomes of the recent zoonotic disease prioritisation exercise, conducted using the One Health Zoonotic Disease Prioritisation Tool [59], to guide future OH investments. In Ethiopia, for example, prioritisation of zoonotic diseases facilitated the formation of a National OH Steering Committee, establishing a framework for coordinated surveillance, control and response across sectors [60]”*

**Comment 8. Examples of Implementation:** Including examples of successful OH strategies from other regions could provide stronger support for the recommendations made in the study.

**Response:** Thank you for the suggestions. We have included examples from other countries to support our recommendations.

Example 1. Page 22, Lines 330 – 334: *“ Addressing this gap requires a comprehensive communication and dissemination strategy for the Bhutan OH Strategic Plan and OH initiatives. An example from rabies control efforts in Chad, Côte d’Ivoire, and Mali has demonstrated how structured communication fostered intersectoral collaboration, enabling strategic policy decisions for effective disease control [17].”*

Example 2. Page 23, Lines 362 – 367: *“These insights collectively highlight the opportunity to integrate OH education into continuous professional development programmes and formal curricula of medical and animal health institutions, as advocated by various organisations [19, 20]. For example, Linder et al. [21] demonstrated that integrating a multidisciplinary curriculum in university settings significantly enhanced student understanding and application of OH concepts.”*

Example 3. Page 25, Lines 394 – 400: *“To address this need, the government may benefit from considering the outcomes of the recent zoonotic disease prioritisation exercise, conducted using the One Health Zoonotic Disease Prioritisation Tool [11], to guide future OH investments. In Ethiopia, for example, prioritisation of zoonotic diseases facilitated the formation of a National OH Steering Committee, establishing a framework for coordinated surveillance, control and response across sectors [18].”*

**Comment 9. Citations and References:** The reference to different figures should be accompanied by brief descriptions to provide context. This would help readers understand the relevance of these figures within the broader study.

**Response**: We have adhered to the figure naming conventions required by the guidelines of *PLOS Global Public Health* and have now included a brief description for each figure.

The figures cited in the manuscript and their descriptions are provided below:

| **Figure** | **Description** |
| --- | --- |
| Figure 1 | Page 6, Lines 121-124: **Figure 1** illustrates the geographical distribution of Bhutan’s 20 districts, providing context for the spatial representation of the respondents in the study |
| Figure 2 | Page 7, Lines 138-140: **Figure 2** illustrates the institutional setup of animal health, human health service centres, BFDA, and the universities across national, regional, district, and sub-district levels, along with the distribution of human resources. |
| S4 Figure | Page 14, Lines 250-251: The frequencies of responses to the knowledge-based questions on OH are presented in **S4 Figure.** |
| S5 Figure | Page 14, Lines 259-260: **S5 Figure** shows the frequencies of responses to attitude-based questions on OH |
| S6 Figure | Page 15, Lines 271-272: **S6 Figure** shows the frequencies of responses to the practice questions of OH. |
| S7 Figure | Page 18, Lines 285-286: The responses to knowledge-based questions on zoonotic disease are illustrated in **S7 Figure.** |
| S8 Figure | Page 18, Lines 299-300: **S8 Figure** displays the frequencies of responses to attitude-based questions on zoonotic diseases. |
| S9 Figure | Page 19, Lines 309-310: The frequencies of responses to practice-based questions on zoonotic diseases are shown in **S9 Figure.** |

**Comment 10. Connection Between Findings:** Strengthening the link between attitudes and

the need for training or support is recommended. For example, discussing how attitudes may influence the demand for OH training could add depth to the findings.

**Response:** Thank you for highlighting this need. We have provided a section that addresses the interplay between training and attitudes, supported by relevant citations.

Page 23, Lines 343 – 348: *“These findings highlight the need for joint training programmes to enhance attitudes towards OH practices. Winer et al. [19] demonstrated a connection between training and attitudes, showing that joint training significantly enhances appreciation for relevance of OH and enthusiasm for collaborative health efforts. Similarly, Edwards et al. [20] provided further support by demonstrating that joint training positively shifts attitudes towards collaboration.”*

# Reviewer 2

## Overall comments

Thank you so much for sharing this cross-sectional, facility-based study that was carried out nationwide through an online questionnaire survey in Bhutan. The topic is very interesting and useful for other countries and regions related to One Health (OH). There are some points that I would like to suggest improving this manuscript's quality.

**Response:** Thank you for reviewing our manuscript and acknowledging its relevance and utility. Your suggestions are invaluable in enhancing our research and ensuring it contributes effectively to the understanding and implementation of One Health approaches, both in Bhutan and globally.

**Overall suggestions**

Please check for grammatical errors, including tenses, citation formats, and spaces between texts throughout the manuscript. Instead of simply referencing previous studies, study methodologies should be more detailed.

**Response:** Thank you. We have thoroughly reviewed the manuscript to correct all grammatical mistakes, standardised the citation formats, and adjusted text spacing as per your suggestions.

**Title:**

**Comment 1.** Should we change to "zoonotic diseases" instead of "zoonotic disease"?

**Response:** We have revised the title as follows:

Page 1, Lines 1 – 2: *“Knowledge, attitude, and practice of One Health and zoonotic diseases among multisectoral collaborators in Bhutan: Results from a nationwide survey”*

**Abstract:**

**Comment 2.** This study assesses the knowledge, attitudes, and practices regarding One Health and zoonotic diseases among key sector professionals to identify gaps and opportunities for enhancing One Health strategies in Bhutan. Please change to "assessed"

**Response:** Thank you very much. We have corrected it accordingly:

Page 2, Lines 15 – 18: *“This study assessed the knowledge, attitudes, and practices regarding One Health and zoonotic diseases among key sector professionals to identify gaps and opportunities for enhancing One Health strategies in Bhutan.”*

**Comment 3.** …..and two-thirds (66%) had above-average practices. Professionals with mid-level job experience ( 6-10 years; AOR = 2.13; 95% CI = 33 1.37-3.30 and 11 to 15 years; AOR = 2.11; 95% CI = 1.31-3.40). Please change to (6-10 years; …)

**Response:** Thank you for noting the oversight. We have corrected the formatting for the age groups in the abstract and addressed similar issues *throughout the manuscript.*

Page 2, Lines 32 – 34: *“Professionals with mid-level job experience (6-10 years; AOR = 2.13; 95% CI = 1.37-3.30 and 11 to 15 years; AOR = 2.11; 95% CI = 1.31-3.40) were more likely to possess above-average knowledge.”*

**Introduction:**

**Comment 4.** Page 3, Line 55: Please revise to "OH concept" has proven …..

**Response:** Thank you for your suggestion. We have updated the sentence as follows:

Page 4, Lines 59 – 60: *“Globally, the OH concept has proven effective in both the detection [21] and responding [22] to zoonotic disease outbreaks.”*

**Comment 5.** Page 3, Line 57: Please provide more information about the drivers and impacts of zoonotic diseases in Bhutan that could be reasons for forming OH, aside from having a list of zoonotic diseases (Page 4, Line 60 – 62). Kindly provide information, especially national health statistics on these zoonotic diseases.

**Response:** Thank you highlighting these critical points essential in our manuscript. We have made further investigation and updated the section with drivers and impact of the zoonotic diseases reported in Bhutan.

Page 4, Lines 61 – 77: *“Bhutan shares many drivers of zoonotic diseases with other countries in South Asia, a region considered one of the global hotspots for emerging and re-emerging diseases [23]. Key factors contributing to the risk of zoonotic disease transmission in Bhutan include the geographical location, increasing agricultural intensification, and rich biodiversity, which foster close human-animal-environment interactions [15]. Additionally, cultural practices such as keeping animals close to homes and allowing them to roam freely, which encourage close human-animal contact, combined with limited veterinary and public health infrastructure, further elevate the risk [24].”*

*“Since 2010, Bhutan has reported 13 outbreaks of highly pathogenic avian influenza, significantly impacting poultry production and rural livelihoods [25-27]. Anthrax is sporadically reported, resulting in deaths in both animals and humans [28]. Rabies remains a significant public health threat, particularly along the southern border with India. For example, 19 human deaths were reported from 2006 to 2023 [29], and approximately 5000 dog bites occur each year [30], requiring the government to spend around USD 142,000 on post-exposure prophylaxis [31]. These recurrent zoonotic outbreaks and their substantial impacts on public health and animal health provided the rationale for Bhutan’s adoption of a OH approach.”*

**Comment 6.** Page 4, Line 70: Please provide more information about having anthrax, rabies, avian influenza, antimicrobial resistance, and leptospirosis[26], as priority diseases.

**Response:** We have included a sentence in the manuscript, that explains the rationale for prioritising anthrax, rabies, avian influenza, antimicrobial resistance, and leptospirosis for the joint development of their prevention and control plan as follows:

Page 5, Lines 83 – 86: *“Major activities undertaken include the joint development of prevention and control plans for anthrax, rabies, avian influenza, antimicrobial resistance, and leptospirosis[16], prioritised based on their potential impact on public health and animal health, and availability of donor funding.”*

**Comment 7.** Page 4, Line 73: please provide more information about sources of external funding.

**Response:** Thank you for pointing the lack of detail on external funding. To provide more information, we have revised the section as follows:

Page 5, Lines 86 – 90: *“Additionally, capacity development programmes, such as training, meetings, and seminars, as well as research into zoonotic diseases, have been conducted through financial and technical support from the World Bank, the World Health Organisation, the World Organisation for Animal Health, Fleming Fund, the European Union, and the Food and Agriculture Organisation of the United Nations [27].”*

**Comment 8.** Page 4, Line 73 – 75: please elaborate on the challenges.

**Response:** We have elaborated the challenges as follows:

Page 5, Lines 90 – 98: *“Despite these initiatives,* *significant challenges hinder OH efforts to control zoonotic diseases in Bhutan, and there is limited national comprehensive scientific inquiry to identify the challenges and possible remedies. Major challenges include inadequate diagnostic capacity due to limited government funding, poorly defined roles of stakeholders, and a lack of formal collaborative mechanisms [15]. Furthermore, surveillance and response measures are weak due to insufficient collaboration among the key stakeholders. Limited data for evidence-based decision-making, due to a scarcity of localised and policy-relevant research, presents an additional challenge [12, 16].”*

**Comment 9.** Page 4, Line 75 – 76: please elaborate on "requires behavioural, attitudinal and institutional change".

**Response:** We have elaborated on the statement and revised the section as follows:

Page 5, Lines 98 – 101: *“Addressing these challenges in OH requires behavioural, attitudinal and, institutional changes [12]. This involves accepting a culture of collaboration and communication among OH stakeholders, shifting attitudes towards shared responsibility for health outcomes and policy alignment across sectors [13].”*

**Comment 10.** Page 4, Line 76 – 78: Please elaborate on these sentences "A Knowledge, Attitude and Practice (KAP) study among key stakeholders can identify opportunities and challenges, providing context-specific information essential to inform and evaluate OH strategies for Bhutan and globally.[27]. Moreover, delete . after globally.

**Response:** Thank you for this feedback. We have revised the section as follows:

Page 5, Lines 102 – 106: *“A Knowledge, Attitude and Practice (KAP) study among key stakeholders can identify opportunities and challenges that influence the success of OH strategies. By assessing the current level of awareness, perceptions and attitudes related to OH and zoonotic diseases among the stakeholders, such a study provides context-specific information that is essential for making targeted interventions [14].”*

**Comment 11.** Page 4 - 5, Line 81 – 85: Are these the current results? If not, please provide citations. If yes, please move to the result section.

**Response:** Thank you for your observation. These are indeed current results, and we apologise for this oversight in placement. We have deleted this section as the information is already included in the result section.

**Comment 12.** Page 5, Line 86: Please change to "This research results are expected to contribute ….."

**Response:** Thank you. We have changed to “expected” and update the section as follows:

Page 6, Lines 109 – 111: *“In addition to providing valuable insights for low- and middle-income countries, the results are expected to contribute to a broader understanding of OH for zoonotic disease management.”*

**Methodology:**

**Comment 13.** Page 5, Line 90 – 99: Please check the Plos format to see whether the "Ethics statement" should be placed here. Please also provide information on how you reached the target respondents for this online survey; for example, how you got their email addresses or shared the online questionnaire.

**Response:** Thank you for the suggestion. We have reviewed the *PLOS Global Public Health* guidelines [32] and confirmed that the “Ethics statement” should indeed be placed within the Materials and methods section. Following this guidance, and after consulting other recent articles [33, 34] published in *PLOS Global Public Health*, we have moved the section to the end of the Materials and methods section (Page 11, Lines 224-233) to ensure consistency with the journal’s format.

Additionally, we have expanded the Materials and methods (Respondent selection) section to include details on the process used to obtain email addresses of eligible participants and to distribute the online questionnaire.

Pages 8 – 9, Lines 160 – 163: *“The research team engaged with institutional heads to brief them on the study objectives and eligibility criteria for participants. Institutional heads subsequently obtained verbal consent from eligible participants and collected a total of 2,783 email addresses from those willing to participate in the study.”*

Furthermore, we have specified on how we distributed the online survey in Materials and methods (Data collection) section as follows:

Page 9, Lines 182 – 183: *“The final questionnaire was distributed through personalised email links using Qualtrics, with settings to anonymise the responses.”*

**Comment 14.** Page 6, Line 106 – 116: Please confirm and provide information on whether they have been involved with OH activities.

**Response:** The respondents in this study were purposively selected from the key OH sectors identified in the Bhutan OH strategic plan. These professionals are actively engaged in various OH activities. The nature and extent of their involvement is influenced by their core mandates within their respective ministries, the OH programs implemented at the national level, and availability of funding.

We have incorporated this clarification in our manuscript as follows:

Page 8, Lines 148 – 150: *“The primary roles of these professionals are defined by the core objectives of their respective parent sectors. They are also integral to the OH system, contributing to various OH activities aligned with national plans and available funding.”*

**Comment 15.** Please revise the sequences of this section per the journal's format. I would suggest the following sequences: Study design, Study locations, Study respondent selections, data collection procedures, and data analysis.

**Response:** Thank you for the valuable suggestion and we have now revised the sequence of sub-titles as suggested.

**Comment 16.** Please add a sub-section to briefly explain Bhutan's health (human and animal) systems and administration. Then, the readers can understand the rationale behind selecting the professions.

**Response:** Thank you for the advice. The manuscript has been updated to include a “Study location” section. This section provides an overview of the animal, and human health systems in Bhutan and highlights their involvement in OH activities.

Pages 7 – 8, Lines 129 – 140 and 145 – 147: *“**Health services are provided by the MoH through primary health centres, districts and general hospitals, and referral hospitals [28]. Animal health services are provided by the MoAL through a network comprising of the National Centre of Animal Health, district veterinary hospitals, and livestock extension offices. Both systems operate under a hierarchical administrative setup, with centralised policy formulation and oversight at the national level while service delivery is managed at regional, district, and sub-district levels. Food safety and regulatory services are provided by the Bhutan Food and Drug Authority (BFDA), with offices at the national and district levels. The CNR, serves as the primary source of human resources for MoAL. The KGUMSB provides medical and healthcare education and training programmes in Bhutan [35]. Figure 2 illustrates the institutional setup of animal health, human health service centres, BFDA, and the universities across national, regional, district, and sub-district levels, along with the distribution of human resources.”*

*“Professionals in these sectors are categorised and posted based on their educational background and their core roles, with national and regional centres employing a higher proportion of professionals with advanced qualifications**.”*

**Comment 17.** Page 7, Line 122: please change "invitees" to "respondents or participants."

**Response:** Thank you for the suggestion. We have replaced “invitees” with “respondents” in Materials and methods (Respondent selection) section as follows:

Page 8, Lines 152 – 154: *“The respondents included medical doctors, health assistants and clinical officers (HA/CO), nurses, laboratory staff, and food regulatory staff from the MoH, as well as veterinarians, para-veterinarians, and veterinary laboratory staff, from the MoAL.”*

**Comment 18.** Page 7, Line 122 – 126: please provide a rationale for selecting these professions for this study.

**Response:** Thank you for the comment. The rationale for selecting these professionals has been clarified in the manuscript’s Material and methods (Respondent selection) section, as follows:

Page 8, Lines 156 – 158: *“These professionals were purposively selected based on their roles within the key OH sectors, where they contribute to OH initiatives and zoonotic disease management at different capacities.”*

**Comment 19.** Page 7, Line 137 – 138: Please provide information about the pre-testing among the respondents. Did the pre-test results were included in the actual analysis?

**Response:** We apologise for the omission of this important clarification in our original manuscript. We have added a sentence to address this to enhance the transparency of our methodology.

Page 9, Lines 181 – 182*: “The pretest respondents were not included in the main survey.”*

**Comment 20.** Page 8, Line 142: how did you obtain their email addresses? Did they agree to share their email addresses with the research team?

**Response:** Thank you for bringing attention to this point again. We would like to note that it has been addressed in our previous response to comment 13, as follows:

Pages 8 – 9, Lines 160 – 163: *“The research team engaged with institutional heads to brief them on the study objectives and eligibility criteria for participants. Institutional heads subsequently obtained verbal consent from eligible participants and collected a total of 2,783 email addresses from those willing to participate in the study.”*

**Comment 21.** Page 8, Line 144: What data collection strategies were used to get a high response rate within a very short period (September 18 to October 13, 2023)? Did you share the online questionnaire with potential respondents during the nationwide meetings?

**Response:** Thank you for this insightful question. To achieve a high response rate within a short period, we implemented several strategies. Before distributing the questionnaire, we coordinated closely with institutional heads and administrators, who encouraged participation by informing eligible respondents about the study and its importance. Once the survey was distributed, heads and administrators sent regular reminders through their respective institutional social media group platforms (e.g., Facebook Messenger, WhatsApp, WeChat and Telegram) to encourage completion. Additionally, we utilised the Qualtrics built-in reminder function to send scheduled reminders with customised messages, emphasizing the importance of participants’ input in enhancing Bhutan’s health system.

While the questionnaire was not distributed during nationwide meetings, our pre-survey communications and consistent follow-up reminders were instrumental in promoting participation.

We have added this information to the manuscript as follows:

Pages 9 – 10, Lines 184 – 187: *“Responses were monitored online throughout this period. Reminders were sent via Qualtrics, and institutional heads were frequently contacted to encourage participation through their respective social media group platforms.”*

**Comment 22.** Page 8, Line 150: Please elaborate on the scoring methods that were similar to the previous studies (ref. 31 and 32).

**Response:** Thank you. We have elaborated on the scoring process in our Materials and methods (Data analysis) section as follows:

Page 10, Lines 192 – 199: *“We adopted a scoring approach similar to those used in Nepal and Uganda [35, 36]. Responses to knowledge-based questions were assigned a score of 1 for correct answers and 0 for incorrect or “Don’t know” responses. Attitude-related questions were scored on a 5-point Likert scale, ranging from 5 for “strongly agree” to 1 for “strongly disagree.” Scores were reversed for negatively stated questions, with 1 assigned to “strongly agree” and 5 to “strongly disagree”. Similarly, practice-related responses were assigned a score of 1 for responses indicating good practices and 0 for responses indicating poor practices.”*

**Comment 23.** Page 8, Line 157: Please elaborate on methods instead of only mentioning, “We followed methods used in other studies [36, 37]”.

**Response:** Thank you. We have provided more clarity to the process of categorising the respondents based on the mean.

Page 10, Lines 200 – 204: *“We categorised the respondents into binary groups, “above-average” and “below-average,” based on their scores relative to the mean, following a methodology commonly applied in KAP studies within health settings [37, 38]. Respondents scoring at or above the mean were categorised as “above-average,” while those scoring below the mean were categorised as “below-average.”*

**Comment 24.** As per the abstract – please elaborate: “Responses from participants were categorized into binary outcomes based on the mean score. Descriptive and summary statistics were performed.”, in the analysis methods of the methodology section.

**Response:** Thank you. We have corrected the sentence in the abstract as:

Page 2, Lines 22 – 23: *“. Respondents were categorised into binary groups based on their knowledge, attitude, and practice scores relative to the mean.”*

Similarly, as stated in response to comment 23, we have elaborated this in the Materials and methods (Data analysis) section as follows:

Page 10, Lines 200 – 204: *“We categorised the respondents into binary groups, “above-average” and “below-average,” based on their scores relative to the mean, following a methodology commonly applied in KAP studies within health settings [37, 38]. Respondents scoring at or above the mean were categorised as “above-average,” while those scoring below the mean were categorised as “below-average.”*

Furthermore, we have elaborated on descriptive and summary statistics in our Materials and methods:

Pages 10, Lines 190 – 192: *“Descriptive and summary statistics were calculated, including frequencies, percentages, proportions and, means for each variable to obtain an overview of the sample characteristics and distributions.”*

**Results:**

**Comment 25.** Page 9, Line 180 – 181: Please check (Table 1) which was separated into different lines.

**Response:** We have corrected the layout, so that “Table 1” now appears on the same line for improved readability ( Page 12, Line 241)

**Comment 26.** Table 1: Please revise the format of the Table.

**Response:** Thank you for your suggestion. We have revised the format of Table 1 to enhance data presentation while ensuring alignment with the guidelines for *PLOS Global Public Health* [39].

The updated table is placed on pages 12 – 14.

**Comment 27.** Page 11, Line 184: Please add – Remark: BFDA, Bhutan Food and Drug Authority

**Response:** We have added “Remark:” before “BFDA, Bhutan Food and Drug Authority” in all the three tables, as follows:

Page 14, Line 244; Page 17, Line 280; Page 21, Line 319: *“Remark: BFDA, Bhutan Food and Drug Authority”*

**Comment 28.** Table 2 - 3: Please revise the tables’ formats and presentation. Also, Table 2, please correct the table headings are correct, which columns are AOR, and which are 95% CI.

**Response:** Thank you for the suggestion. We have revised the format and presentation of Tables 2 and 3 in accordance to the journal’s guidelines [39].

Furthermore, the misplaced column headings in Table 2 have been corrected to accurately indicate the AOR and 95% CI.

The revised versions of tables 2 and 3 are placed on pages 16 – 17 and 20 – 21 respectively.

**Discussions:**

**Comment 29.** Page 22, Line 257: Please change to “demonstrated”.

**Response:** Thank you for your suggestions. We have changed the term to “demonstrated” and revised the sentence as follows:

Page 22, Lines 324 – 325: *“At least half of the respondents demonstrated above-average knowledge, attitudes, and practices regarding OH and zoonotic diseases prevention and control.”*

**Comment 30.** Page 22, Line 257 – 259: Please paraphrase these sentences.

**Response:** The sentence has been divided into two and revised as follows:

Page 22, Lines 324 – 326: *“At least half of the respondents demonstrated above-average knowledge, attitudes, and practices regarding OH and zoonotic diseases prevention and control. However, several gaps were identified, which are discussed in the following sections.”*

**Comment 31.** Page 23, Line 285: Please provide study locations of the previous studies (ref 32 and 44) instead of only mentioning the “similar studies”.

**Response:** We have revised the sentence to include the specific study locations for references 32 and 44, providing clearer context for the comparison with similar studies.

Page 23, Lines 358 – 360: *“Additionally, the prevalence of better knowledge among higher qualified respondents supports the positive correlation between higher education levels and increased knowledge, as observed in similar studies conducted in Nepal and Zambia [36, 40].”*

**Comment 32.** Page 23, Line 288 – 289: Please elaborate on “the needs” in specific activities and curricula, such as knowledge of topics that should be improved, such as clinical, epidemiology, OH, or else?

**Response:** Thank you for the suggestion. We have elaborated the needs of training in the recommendation (Develop OH workforce) section as follows:

Page 27, Lines 452 – 460: *“Short-term strategies should focus on joint in-service training on foundational OH concepts such as collaboration and communication, progressing to skills in zoonotic disease management, field epidemiology, laboratory technologies, and policy formulation. Long-term strategies should prioritise integrating OH education into CNR and KGUMSB curricula, ensuring competencies among future OH professionals. Curriculum development can draw on models such as those developed by the One Health Central and Eastern Africa (OHCEA) network [7] and the FAO [8]. Such efforts may be regularly evaluated using frameworks such as the OH core competencies [9] to identify the areas of improvement and enhance efficiency.”*

**Comment 33.** Page 24, Line 304 – 308: Please elaborate on the results of the previous studies compared with the current findings.”

**Response:** Thank you. We have compared our result with results from a previous study, and revised the section as follows:

Page 24, Lines 383 – 388: *“Our findings indicate a lower level of attitudes toward zoonotic diseases among medical doctors, contrasting with findings of Mligo et al. [41], who reported a positive attitude towards brucellosis among medical officers in rural Tanzania. This difference may be attributed to factors such as distinct geographical context, the zoonotic diseases examined, and the differences in the healthcare systems in the two studies.”*

**Comment 34.** Page 24, Line 321 – 324: Please elaborate on the rationale for the limited number of studies in Bhutan that could be associated with the current results, including the OH implementation in the country.

**Response:** Thank you for this insightful comment. We have expanded the section to elaborate on the factors contributing to the limited number of studies and their association with our study results:

Page 25, Lines 407 – 412: *“Furthermore, our study revealed that the majority of respondents have not engaged in zoonotic disease research. This gap likely results from inadequate funding and training, as indicated by responses highlighting the need for increased international support, sectoral funding, and training opportunities. These challenges align with findings from South-Asia, where limited funding and training are recognised as primary barriers to advancing OH research [12].”*

**Comment 35.** Any strengths of this current study?

**Response:** We have now included a section on the study’s strengths, as follows:

Page 26, Lines 426 – 435: *“The main strength of our study was its nationwide coverage, across multiple key OH sectors at multiple levels. This coverage enabled a comprehensive assessment of knowledge, attitudes, and practices of key OH stakeholders across Bhutan. The sample size significantly exceeded our initial target, enhancing the statistical validity and generalizability of our results. Additionally, our study benefited from a rigorous methodology, employing a validated and pre-tested questionnaire, developed with expert input.*

*Furthermore, the study’s findings were not only descriptive but also solution-oriented, with actionable recommendations. Framed within a global OH context, the study offers insights that contribute to strengthening OH initiatives, particularly in resource-limited settings, enhancing its relevance for Bhutan and other similar contexts.”*

**Comment 36.** As mentioned, the limitation of the study was the exclusion of professionals from the environmental sector. Kindly elaborate on additional limitations of other selection biases, such as selecting only those who have email addresses or were pre-selected. As the questionnaire was quite lengthy, how could you avoid missing responses per question? Did you include all the missing items in the analysis?

**Response**: Thank you for the suggestion. We have elaborated on the additional limitations as recommended.

Page 26, Lines 417 – 425: *“A notable limitation of this study was the exclusion of professionals from the environmental sector, due to government restructuring that occurred shortly before the study commenced. This exclusion may have led to the omission of perspectives critical to the environmental aspects of the OH approach. The survey’s reliance on email distribution likely excluded professionals without regular access to digital communication tools, potentially skewing the dataset toward respondents from technologically equipped areas. Although all the survey questions were mandatory, effectively preventing missing data, 33 (3.3%) incomplete surveys were excluded from the analysis. This exclusion may have resulted in loss of valuable insights from respondents who partially engaged with the survey.”*

**Comment 37.** Please have a separate “recommendation section” and provide in-depth plans and strategies that policymakers should consider to improve the OH structure and implementation activities.

**Response:** Thank you for the suggestion. We have created a separate recommendation section, as outlined in response to comment 4 from reviewer1. The recommendations are placed on pages 26 – 28 of the revised manuscript.

***

**Reference**

1.	World Health Organization. Joint Tripartite (FAO, OIE, WHO) and UNEP Statement 2021 [cited 2024 October 24]. Available from: <https://www.who.int/news/item/01-12-2021-tripartite-and-unep-support-ohhlep-s-definition-of-one-health>.

2.	One Health Global Network. One Health: a concept that became an approach and then a movement. 2015 [cited 2024 October 24]. Available from: <https://www.onehealthglobal.net/what-is-one-health/>.

3.	Szumilas M. Explaining odds ratios. Journal of the Canadian academy of child and adolescent psychiatry. 2010;19(3):227.

4.	Gardner MJ, Altman DG. Confidence intervals rather than P values: estimation rather than hypothesis testing. BMJ. 1986;292(6522):746-50. doi: 10.1136/bmj.292.6522.746.

5.	Yopa DS, Massom DM, Kiki GM, Sophie RW, Fasine S, Thiam O, et al. Barriers and enablers to the implementation of one health strategies in developing countries: a systematic review. Frontiers in public health. 2023;11:1252428.

6.	World Health Organization. WHO Strategic communications framework for effective communications 2017 [cited 2024 October 2]. Available from: <https://www.who.int/docs/default-source/documents/communicating-for-health/communication-framework.pdf>.

7.	Amuguni H, Bikaako W, Naigaga I, Bazeyo W. Building a framework for the design and implementation of One Health curricula in East and Central Africa: OHCEAs One Health Training Modules Development Process. One Health. 2019;7:002-2. Epub 20180908. doi: 10.1016/j.onehlt.2018.08.002. PubMed PMID: 30569012; PubMed Central PMCID: PMCPMC6288412.

8.	Food and Agriculture Organization. One Health training manual 2024 [cited 2024 October 17]. Available from: <https://openknowledge.fao.org/server/api/core/bitstreams/a69a382a-2dc9-45d1-8eef-723abe088562/content>.

9.	Laing G, Duffy E, Anderson N, Antoine-Moussiaux N, Aragrande M, Luiz Beber C, et al. Advancing One Health: Updated core competencies. CABI One Health. 2023. doi: 10.1079/cabionehealth.2023.0002.

10.	Ministry of Health Bhutan, Ministry of Agriculture and Forest Bhutan, World Health Organization, World Organization for Animal Health. Kingdom of Bhutan-IHR-PVS National Bridging Workshop-March 2018 2018 [cited 2024 14 November]. Available from: <https://rr-asia.woah.org/app/uploads/2020/05/ihr-bhutan-roadmap.pdf>.

11.	U.S. Centers for Disease Control and Prevention. One Health Zoonotic Disease Prioritization (OHZDP) 2024 [cited 2024 November 13]. Available from: <https://www.cdc.gov/one-health/php/prioritization/index.html>.

12.	McKenzie JS, Dahal R, Kakkar M, Debnath N, Rahman M, Dorjee S, et al. One Health research and training and government support for One Health in South Asia. Infect Ecol Epidemiol. 2016;6:33842. Epub 20161129. doi: 10.3402/iee.v6.33842. PubMed PMID: 27906123; PubMed Central PMCID: PMCPMC5131453.

13.	Chaddock M. Academic Veterinary Medicine and One Health Education: It Is more than Clinical Applications. Journal of Veterinary Medical Education. 2012;39(3):241-6. doi: 10.3138/jvme.0612-062. PubMed PMID: 22940444.

14.	Ana Dinis, Cláudia Augusto, Filipe Maximiano Alves de Sousa, Alison Burrell, Angela Bearth, Isabel Silva, et al. The use of Knowledge, Attitude and Practice (KAP) surveys in One Health studies: a scoping review: The Society for Veterinary Epidemiology and Preventive Medicine; 2023 [cited 2024 November 18]. Available from: <https://svepm.org.uk/wp-content/uploads/2024/07/Carmo-The-use-of-KAP-surveys-in-One-Health-studies-a-scoping-review.pdf>.

15.	Government of Bhutan. Bhutan One Health Strategic Plan 2018-2023. 2018.

16.	Dahal R, Upadhyay A, Ewald B. One Health in South Asia and its challenges in implementation from stakeholder perspective. Vet Rec. 2017;181(23):626. Epub 20171030. doi: 10.1136/vr.104189. PubMed PMID: 29084821.

17.	Léchenne M, Traore A, Hattendorf J, Kallo V, Oussiguere A, Tetchi M, et al. Increasing rabies data availability: The example of a One Health research project in Chad, Côte d'Ivoire and Mali. Acta tropica. 2021;215:105808.

18.	Erkyihun GA, Gari FR, Edao BM, Kassa GM. A review on One Health approach in Ethiopia. One health outlook. 2022;4(1):8-. doi: 10.1186/s42522-022-00064-z.

19.	Winer JN, Nakagawa K, Conrad PA, Brown L, Wilkes M. Evaluation of medical and veterinary students' attitudes toward a one health interprofessional curricular exercise. Journal of Interprofessional Care. 2015;29(1):49-54.

20.	Edwards JC, Walsum Kv, Sanders CW, Fossum TV, Sadoski M, Bramson R, et al. Attitudes of veterinary medical students and medical students toward collaborative learning: an experiment. Journal of veterinary medical education. 2004;31(1):77-9.

21.	Innes GK, Lambrou AS, Thumrin P, Thukngamdee Y, Tangwangvivat R, Doungngern P, et al. Enhancing global health security in Thailand: Strengths and challenges of initiating a One Health approach to avian influenza surveillance. One Health. 2022;14:100397. doi: <https://dx.doi.org/10.1016/j.onehlt.2022.100397>.

22.	Buttigieg SC, Savic S, Cauchi D, Lautier E, Canali M, Aragrande M. Brucellosis control in Malta and Serbia: a One Health evaluation. Frontiers in Veterinary Science. 2018;5(July):147.

23.	Tennakoon T, Wijesundera K. Emerging and Re‐emerging Zoonoses in South Asia: Challenges of One Health. One Health: Human, Animal, and Environment Triad. 2023:273-85.

24.	Durrance-Bagale A, Rudge JW, Singh NB, Belmain SR, Howard N. Drivers of zoonotic disease risk in the Indian subcontinent: A scoping review. One Health. 2021;13:100310.

25.	Tenzin T, Wangdi C, Rai PB. Biosecurity survey in relation to the risk of HPAI outbreaks in backyard poultry holdings in Thimphu city area, Bhutan. BMC veterinary research. 2017;13:1-9.

26.	Marinova-Petkova A, Franks J, Tenzin S, Dahal N, Dukpa K, Dorjee J, et al. Highly pathogenic reassortant avian influenza A (H5N1) Virus Clade 2.3. 2.1 a in Poultry, Bhutan. Emerging Infectious Diseases. 2016;22(12):2137.

27.	Zinsstag J. One health : the theory and practice of integrated health approaches. Second edition. ed. Wallingford, Oxfordshire, UK ;: CABI; 2021.

28.	Ministry of Health Bhutan. Guidelines for Preparedness, Surveillance and Control of

Anthrax in Human and Animals in Bhutan. 2013. p. 1.

29.	Dorji T, Lamichaney J, Gyaltshen C, Lungten L, Dhakal GP, Dorjee S, et al. Human rabies encephalomyelitis in the background of rabies outbreak in animals in Gelephu, Bhutan, 2023: a case report. Infectious Diseases of Poverty. 2023;12(1):94.

30.	Ministry of Agriculture and Forests Bhutan. National Rabies Prevention and Control Plan. 2 ed2017. p. 5.

31.	Penjor K, Tenzin T, Jamtsho RK. Determinants of health seeking behavior of animal bite victims in rabies endemic South Bhutan: a community-based contact-tracing survey. BMC Public Health. 2019;19(1):237. doi: 10.1186/s12889-019-6559-x.

32.	PLOS Global Public Health. Submission guidelines n.d. [cited 2024 November 7]. Available from: <https://journals.plos.org/globalpublichealth/s/submission-guidelines>.

33.	Banke-Thomas A, Lewis O, Duduyemi A, Ogunyemi O, Nadan T. Motivations for and experiences of childbirth abroad amongst Nigerian women: A qualitative study. PLOS Global Public Health. 2024;4(9):e0003737. doi: 10.1371/journal.pgph.0003737.

34.	Ssali PW, Kintu TM, Karungi I, Namuyaba AK, Kyagambiddwa T, Namaseruka R, et al. “If you find that I am HIV positive, don’t tell me”: Exploring the barriers and recommendations for HIV prevention services utilization among youth in rural southwestern Uganda. PLOS Global Public Health. 2024;4(9):e0002555. doi: 10.1371/journal.pgph.0002555.

35.	Subedi D, Gautam A, Sapkota D, Subedi S, Sharma S, Abdulkareem M, et al. Knowledge and perception of veterinary students on One Health: A first nationwide multi-institutional survey in Nepal. International Journal of One Health. 2022:34-42. doi: 10.14202/ijoh.2022.34-42.

36.	Monje F, Erume J, Mwiine FN, Kazoora H, Okech SG. Knowledge, attitude and practices about rabies management among human and animal health professionals in Mbale District, Uganda. One Health Outlook. 2020;2:24. Epub 20201214. doi: 10.1186/s42522-020-00031-6. PubMed PMID: 33829139; PubMed Central PMCID: PMCPMC7993504.

37.	Abuduxike G, Acar Vaizoglu S, Asut O, Cali S. An Assessment of the Knowledge, Attitude, and Practice Toward Standard Precautions Among Health Workers From a Hospital in Northern Cyprus. Saf Health Work. 2021;12(1):66-73. Epub 20200911. doi: 10.1016/j.shaw.2020.09.003. PubMed PMID: 33732531; PubMed Central PMCID: PMCPMC7940130.

38.	Limbu DK, Piryani RM, Sunny AK. Healthcare workers' knowledge, attitude and practices during the COVID-19 pandemic response in a tertiary care hospital of Nepal. PLoS One. 2020;15(11):e0242126. Epub 20201106. doi: 10.1371/journal.pone.0242126. PubMed PMID: 33156873; PubMed Central PMCID: PMCPMC7647104.

39.	PLOS Global Public Health. Tables n.d. [cited 2024 November 10]. Available from: <https://journals.plos.org/globalpublichealth/s/tables>.

40.	Daka V, Mukosha M, Matafwali SK, Mudenda S, Phiri AM. Knowledge and practices of toxoplasmosis among healthcare workers at two large referral hospitals in Zambia: Implications on the One Health Approach. PLOS Glob Public Health. 2023;3(8):e0002235. Epub 20230815. doi: 10.1371/journal.pgph.0002235. PubMed PMID: 37582063; PubMed Central PMCID: PMCPMC10426967.

41.	Mligo BJ, Sindato C, Yapi RB, Mathew C, Mkupasi EM, Kazwala RR, et al. Knowledge, attitude and practices of frontline health workers in relation to detection of brucellosis in rural settings of Tanzania: a cross-sectional study. One Health Outlook. 2022;4(1):1. Epub 20220104. doi: 10.1186/s42522-021-00056-5. PubMed PMID: 34983693; PubMed Central PMCID: PMCPMC8725462.

*********
